# Management of Discolored Endodontically Treated Maxillary Central Incisors With Nonsurgical Retreatment and Internal Bleaching: A Case Report

**DOI:** 10.7759/cureus.113145

**Published:** 2026-07-22

**Authors:** Hazal Faiz Arslanparcasi, Mehmet Ali Arslanparcasi

**Affiliations:** 1 Endodontics, Faculty of Dentistry, Harran University, Sanliurfa, TUR; 2 Dental Prosthesis Technology, Harran University Vocational School of Health Services, Sanliurfa, TUR; 3 Pediatric Dentistry, Faculty of Dentistry, Harran University, Sanliurfa, TUR

**Keywords:** endodontic retreatment, internal bleaching, maxillary central incisor, nonvital tooth bleaching, tooth discoloration

## Abstract

Tooth discoloration in endodontically treated anterior teeth represents a clinically significant problem, particularly in cases with high esthetic expectations. When pain, percussion sensitivity, or radiographic findings accompany such teeth, the existing endodontic status should be reassessed before esthetic treatment. This case report presents the clinical management of a 28-year-old patient who presented with pain, percussion sensitivity, and marked coronal discoloration in previously endodontically treated maxillary central incisors, teeth #11 and #21. After removal of the existing root canal filling materials, the root canals were reshaped, an irrigation protocol was performed, and calcium hydroxide was placed as an intracanal medicament. After resolution of the symptoms, the root canals were obturated with a bioceramic-based root canal sealer and gutta-percha. Following the placement of a cervical barrier, internal bleaching was performed. At the end of treatment, the patient’s symptoms had resolved, and a marked improvement in anterior esthetics was observed. This case demonstrates that, in symptomatic and discolored endodontically treated anterior teeth, initial infection control followed by conservative esthetic rehabilitation with internal bleaching may represent a clinically appropriate approach.

## Introduction

Tooth discoloration occurring in endodontically treated teeth is an important clinical problem that may affect patients’ quality of life, particularly in the anterior region, where esthetic expectations are high [1]. Discoloration in non-vital teeth may develop due to various factors, including the penetration of hemoglobin degradation products released after pulpal necrosis into dentinal tubules, intrapulpal hemorrhage, irrigating solutions and root canal filling materials used during endodontic treatment, and discoloration associated with coronal restorations over time. During this process, changes in the optical properties of dentin and a reduction in its natural translucency may cause the discoloration to become progressively more evident [2].

On the other hand, the presence of clinical findings such as pain, percussion sensitivity, or periapical pathology in previously root canal-treated teeth may suggest failure of the existing endodontic treatment [3]. In such cases, nonsurgical endodontic retreatment is considered the first treatment option for tooth preservation, as it aims to reclean, reshape, and effectively disinfect the infected root canal system [4]. The primary goal of endodontic retreatment is to eliminate infection from the root canal system, promote healing of the periapical tissues, and maintain the long-term functional success of the tooth [5].

After achieving endodontic success, rehabilitation of discolored non-vital teeth located in the esthetic region constitutes an integral part of treatment. Currently, minimally invasive approaches aimed at maximizing the preservation of dental hard tissues have gained prominence, and internal bleaching is widely used as an effective, safe, and conservative method for managing discoloration in non-vital teeth [6]. When performed with appropriate indications, proper material selection, and adherence to safety protocols, particularly the placement of a cervical barrier, internal bleaching can make an important contribution to restoring the natural esthetic appearance of the tooth without the need for more invasive restorative procedures [7].

This case report aims to present the clinical management and treatment outcomes of nonsurgical endodontic retreatment followed by internal bleaching in previously endodontically treated maxillary central incisors presenting with symptomatic apical findings and marked coronal discoloration.

## Case presentation

A 28-year-old male patient presented to our clinic with complaints of pain and discoloration in the maxillary central incisors (teeth #11 and #21). The patient’s medical history revealed that endodontic treatment had previously been performed on the affected teeth. Clinical examination showed marked coronal discoloration in both teeth (Figure 1). In addition, both teeth exhibited percussion sensitivity, and the patient reported spontaneous pain as well as pain on pressure. 

**Figure 1:**
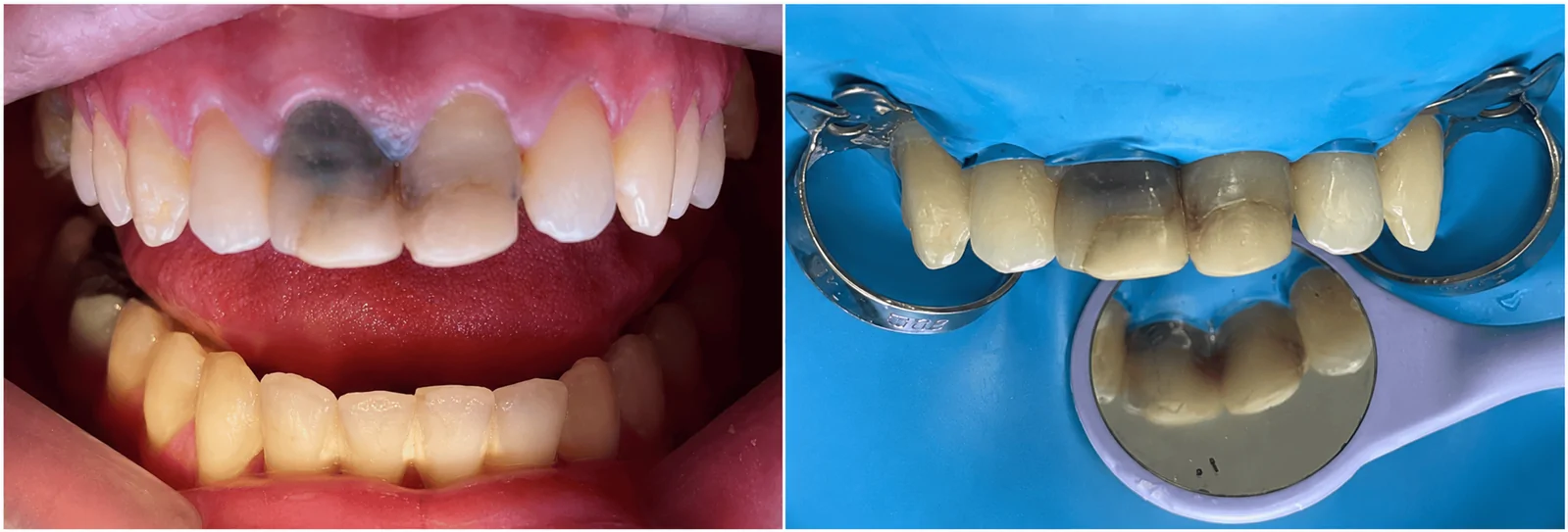
Preoperative intraoral clinical views of teeth #11 and #21.

Radiographic examination revealed that the existing root canal fillings in both teeth terminated markedly short of the radiographic apices, leaving the apical portions inadequately obturated. No distinct periapical radiolucency was detected in either tooth (Figure 2). Based on the patient’s history and the clinical and radiographic findings, the pulpal status of both teeth was classified as previously treated, and the apical diagnosis was symptomatic apical periodontitis. Considering the inadequate obturation together with spontaneous pain and percussion sensitivity, nonsurgical endodontic retreatment of both teeth, followed by internal bleaching of tooth #11, was planned.

**Figure 2:**
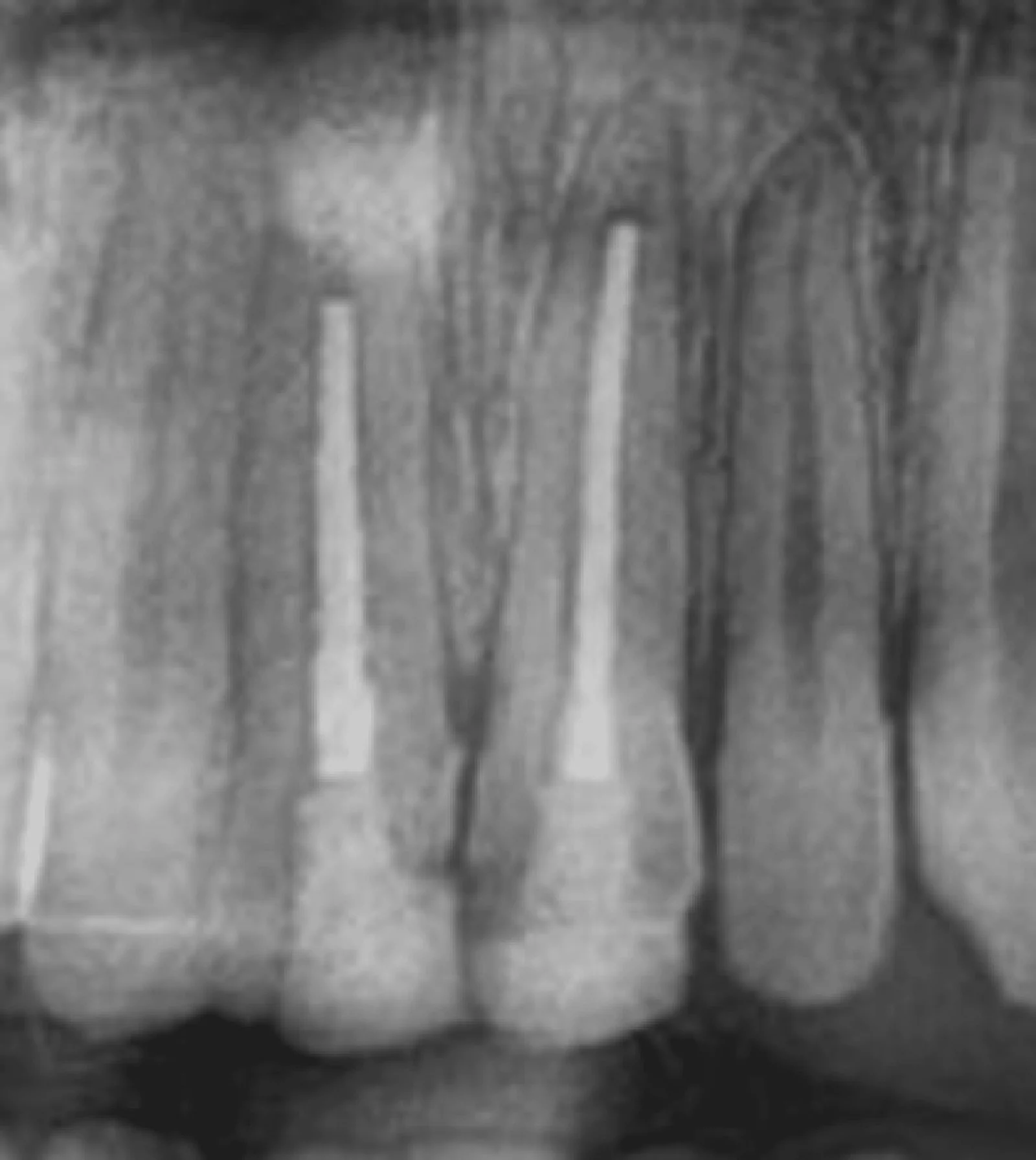
Preoperative periapical radiograph showing inadequate root canal fillings in teeth #11 and #21, terminating markedly short of the radiographic apices. No distinct periapical radiolucency was detected in either tooth.

During the first session, treatment was performed under rubber dam isolation. The existing root canal filling materials were removed using retreatment files (D Perfect Super System Retreat One, Dental Perfect, Shenzhen, China) (Figure 3). The working length was determined using an electronic apex locator and confirmed with a periapical radiograph. The root canals were shaped using the Orodeka Plex V file system (Jining Deca Medical Equipment Co., Ltd., Jining, Shandong, China) to a final apical preparation size of 40/.04. During preparation, 5.25% sodium hypochlorite (NaOCl) was used as the irrigating solution. After completion of the preparation, 17% ethylenediaminetetraacetic acid (EDTA) was applied to remove the smear layer, followed by final irrigation with 5.25% NaOCl. The final NaOCl irrigation was sonically activated using the EDDY system (VDW, Munich, Germany). After the canals were dried, calcium hydroxide (ImiCal, Imicryl, Turkey) was placed as an intracanal medicament, and the access cavities were sealed with a temporary restorative material. The patient was recalled 15 days later.

**Figure 3:**
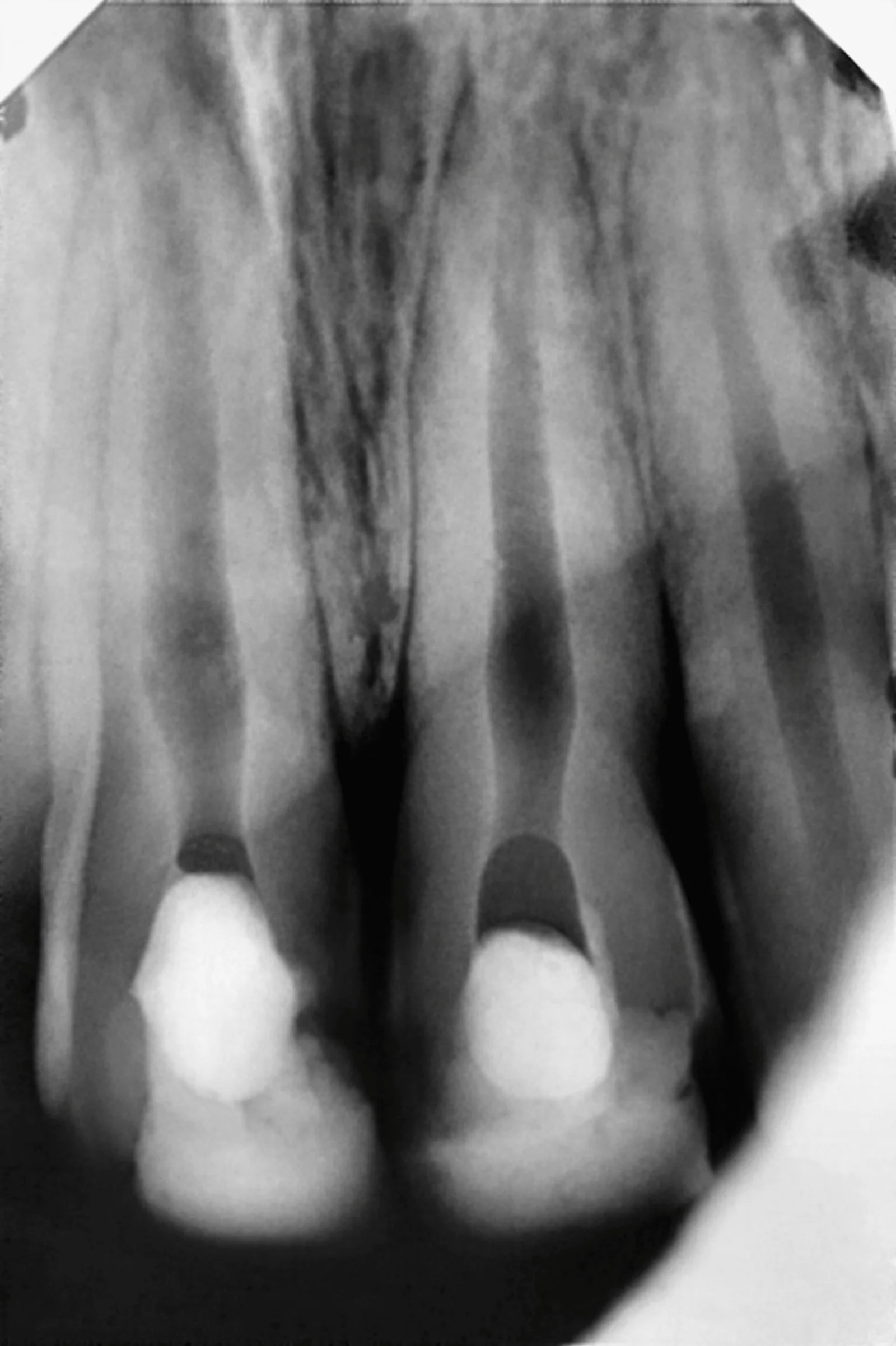
Periapical radiograph obtained after removal of the existing root canal filling materials from teeth #11 and #21 and before canal reshaping.

During the second session, the patient’s spontaneous pain had completely resolved, and no percussion sensitivity was detected. Based on the clinical findings, it was decided to complete the root canal treatment. Under rubber dam isolation, the root canals were obturated with gutta-percha and a bioceramic-based root canal sealer (Bio-C Sealer, Angelus, Londrina, Brazil). After obturation, the gutta-percha was removed approximately 2-3 mm apical to the cementoenamel junction. To limit the penetration of the bleaching agent into the root canal system and cervical periodontal tissues, the canal orifices were sealed with glass ionomer cement, creating a protective cervical barrier whose coronal margin was positioned at the level of the cementoenamel junction. The existing defective coronal restoration was completely removed before internal bleaching was initiated.

Internal bleaching was then performed only on tooth #11, which exhibited more pronounced discoloration. A bleaching agent (Opalescence Endo, Ultradent Products, South Jordan, UT, USA) was placed once into the pulp chamber (Figure 4), and the cavity was sealed with a temporary restorative material. The patient was recalled three days later.

**Figure 4:**
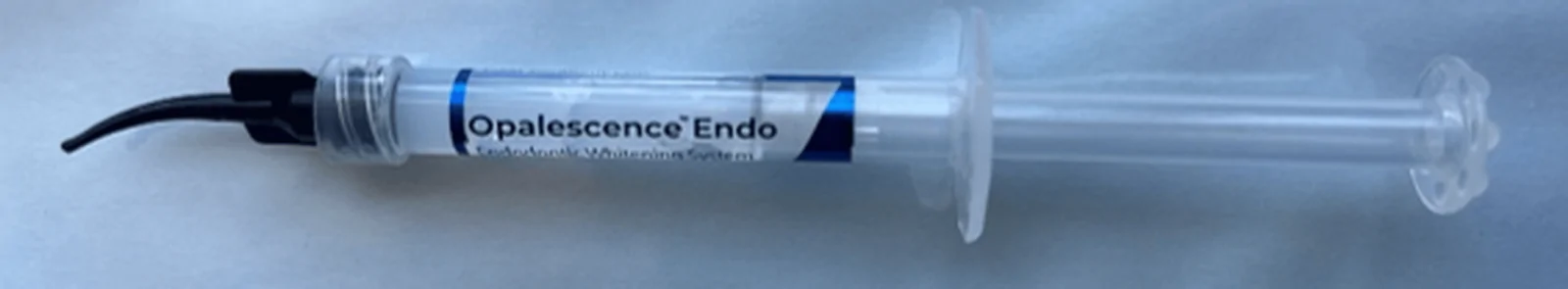
Bleaching agent used for internal bleaching.

At the three-day follow-up appointment, the bleaching agent was removed, and the pulp chamber was thoroughly cleaned with copious irrigation. The bleaching agent was not reapplied. As satisfactory color improvement was achieved after the initial application, no additional bleaching cycle was required; however, repeat application would have been considered if the esthetic outcome had been insufficient. Considering that the residual effects of bleaching agents may adversely affect adhesive systems and the polymerization of composite resins, the definitive restoration was postponed, and the patient was scheduled for an appointment one week later.

At the final session, after application of the adhesive system, the coronal restoration was completed with a composite resin material (Estelite Sigma Quick A2, Tokuyama Dental, Tokyo, Japan). At the end of treatment, the patient was asymptomatic, and a marked improvement in anterior esthetics was observed. The postoperative clinical photograph and periapical radiograph were obtained at this final appointment, approximately one month after the initial presentation (Figures 5, 6). The radiograph was obtained to document the completed root canal fillings and the early postoperative status; periapical bony healing was not evaluated within this short interval.

**Figure 5:**
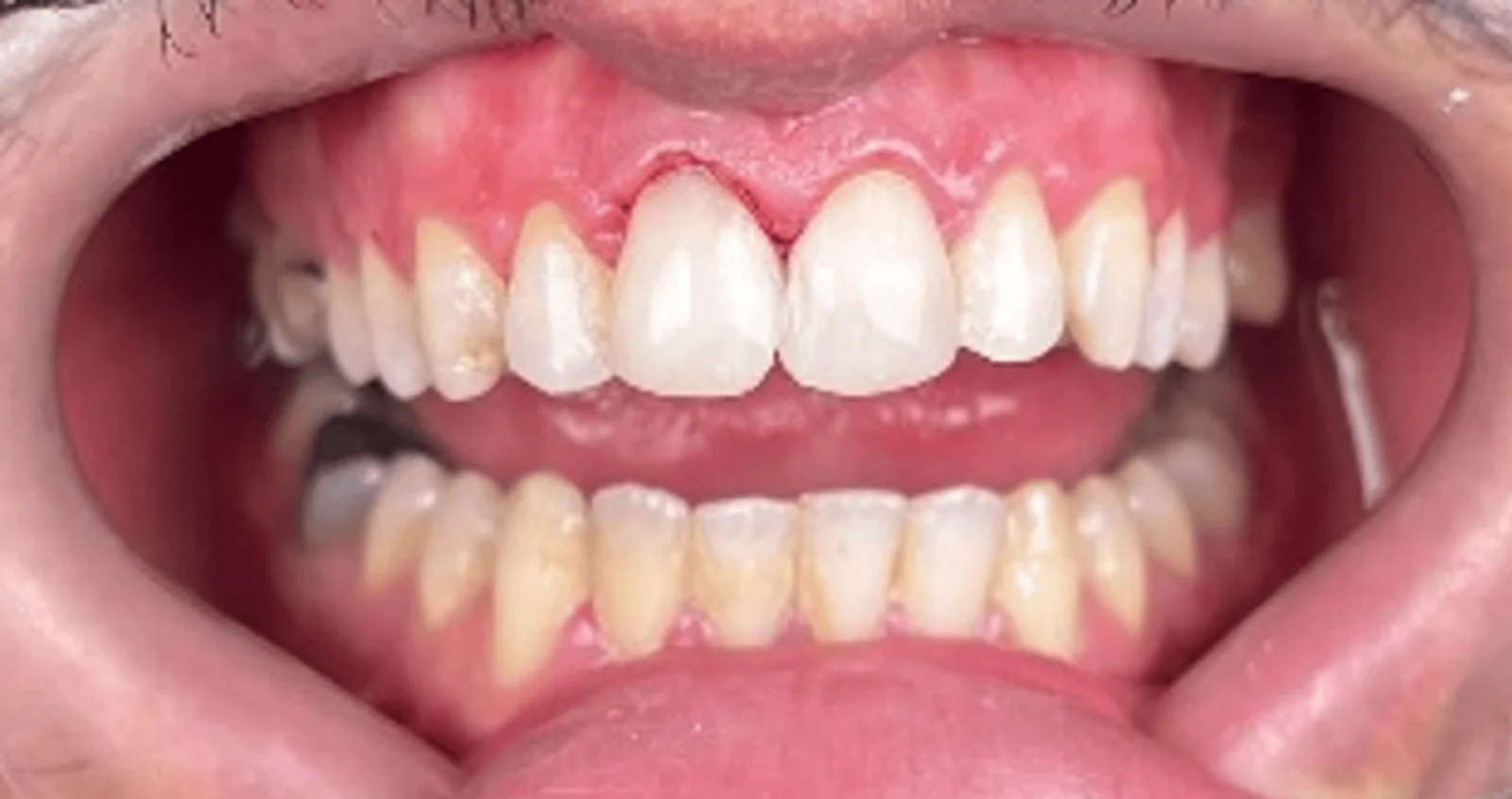
Postoperative intraoral clinical view obtained at the final treatment appointment, approximately one month after the initial presentation.

**Figure 6:**
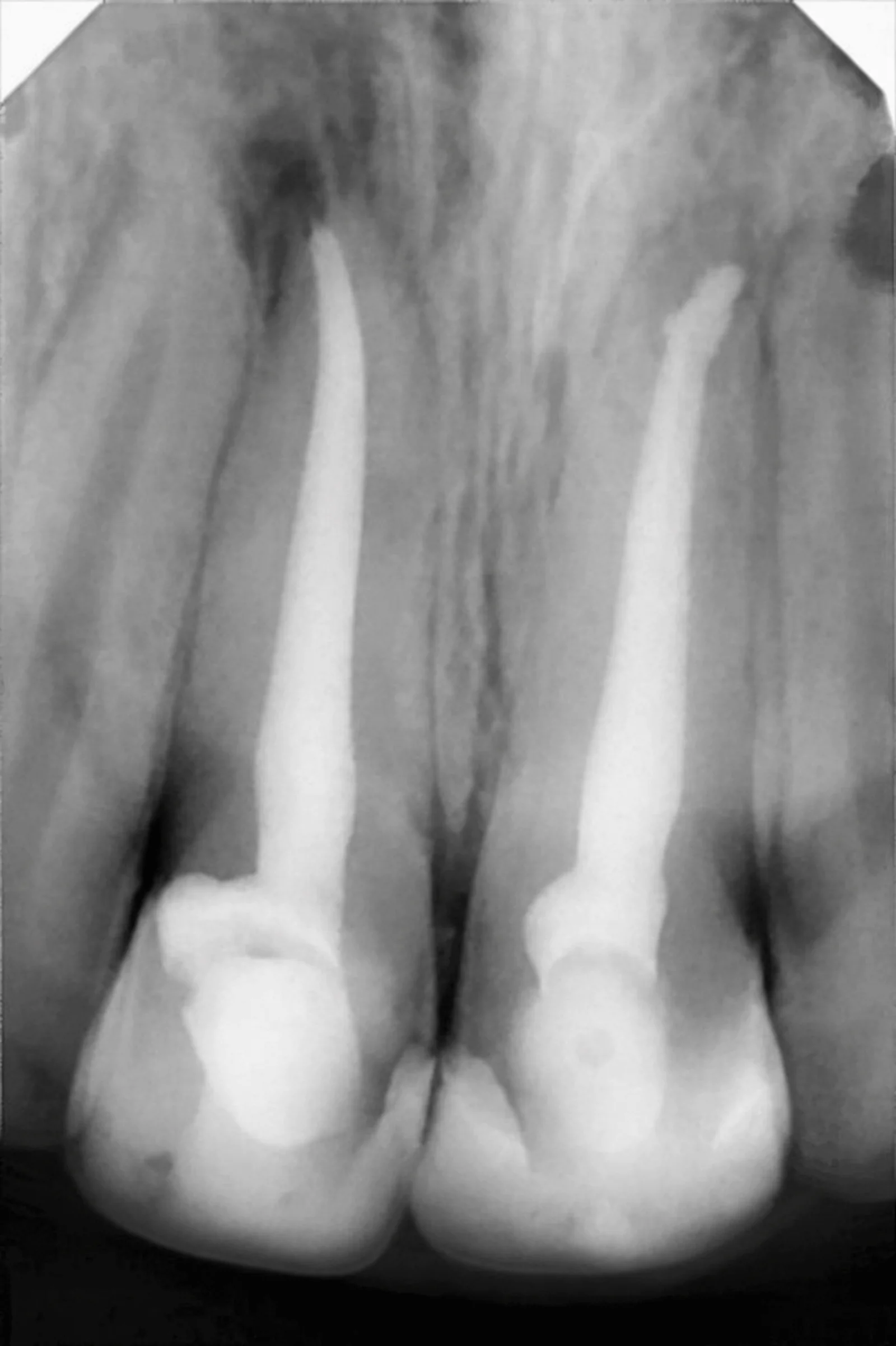
Early postoperative periapical radiograph obtained at the final treatment appointment, approximately one month after the initial presentation.

## Discussion

The coexistence of symptomatic failure and esthetic discoloration in endodontically treated teeth represents a clinically complex situation requiring careful treatment planning. In such cases, the primary objective is to eliminate infection from the root canal system and promote biological healing of the periapical tissues [8]. Subsequently, the use of minimally invasive approaches, such as internal bleaching, to meet esthetic demands is consistent with contemporary endodontic and restorative treatment principles [7]. The present case is important in demonstrating the clinical application of this two-stage approach.

Nonsurgical endodontic retreatment is a fundamental treatment approach for preserving teeth with failed root canal treatment. This procedure aims to remove infected filling materials, reshape the root canal system, and achieve effective disinfection to support biological healing of the tissues [9]. In the present case, nonsurgical endodontic retreatment was performed based on the existing symptoms and radiographic findings, and a favorable clinical outcome was achieved, as the patient became asymptomatic by the second session.

Calcium hydroxide is widely preferred as an intracanal medicament because of its antibacterial effect associated with its high pH, its ability to inactivate endotoxins, and its potential to reduce the residual microbial load within the root canal system [10]. It is of particular clinical importance in retreatment cases because it may contribute to additional disinfection of the canal system [11]. In the present case, the resolution of clinical symptoms after the interappointment use of calcium hydroxide paste was considered a favorable clinical finding associated with the disinfection and medication protocol applied.

The use of bioceramic-based root canal sealers for obturation has become increasingly common in recent years because of their biocompatibility, low solubility, chemical bonding to dentin, and ability to induce hydroxyapatite formation [12]. Their ability to set in moist environments and provide long-term sealing may contribute positively to clinical success [13]. In the present case, the absence of clinical symptoms after obturation with gutta-percha and a bioceramic-based root canal sealer was consistent with the favorable clinical outcome of the applied treatment protocol.

Internal bleaching is considered a minimally invasive treatment alternative that preserves dental hard tissue in the management of discoloration developing after endodontic treatment, particularly in non-vital anterior teeth [7]. Through the diffusion of the bleaching agent placed into the pulp chamber, discoloration that has penetrated into the dentinal tubules is oxidized, thereby improving the esthetic appearance of the tooth [14]. In the present case, a clinically satisfactory esthetic improvement was achieved within a short period following internal bleaching. In the present case, the discoloration was considered multifactorial. During clinical examination, remnants of the previous root canal sealer and gutta-percha were observed within the pulp chamber, together with marginal leakage, secondary caries, and marked dentinal discoloration beneath the defective coronal restoration. These findings suggested that residual endodontic materials and microleakage associated with the deteriorated coronal restoration may have contributed to the discoloration [1,2]. Removal of these materials and the defective restoration during retreatment eliminated potential local sources of staining, whereas internal bleaching was performed to address the remaining intrinsic dentinal discoloration. Therefore, the observed esthetic improvement should be attributed to the combined approach involving retreatment, removal of the defective restoration, and internal bleaching rather than to bleaching alone.

A critical point in internal bleaching procedures is the creation of an effective cervical barrier. Otherwise, diffusion of bleaching agents into the periodontal tissues may increase the risk of external cervical resorption [15]. Therefore, placement of a glass ionomer cement barrier at the level of the cementoenamel junction is important for the biological safety of the treatment, and this principle was followed in the present case.

Residual oxygen released after bleaching is known to adversely affect the polymerization of adhesive systems and the bond strength of composite resins. Therefore, a waiting period before definitive restoration is recommended [16]. In the present case, this biochemical effect was taken into consideration, and the restorative procedure was postponed for one week before the adhesive system and composite restoration were completed.

If the esthetic outcome after internal bleaching is insufficient, direct composite restorations, laminate veneers, or full-coverage crowns may be considered as alternative treatment options. Direct composite restorations may provide a more conservative approach when sufficient sound tooth structure remains and only limited color or form correction is required. Laminate veneers may be preferred when additional masking of persistent discoloration and more extensive esthetic modification are required while adequate tooth structure is preserved. Full-coverage crowns may be considered in cases of severe persistent discoloration accompanied by extensive coronal tissue loss or large existing restorations. Treatment selection should be based on the remaining tooth structure, pre-existing restorations, severity of discoloration, and the patient’s esthetic expectations [17].

The main limitation of this case report is that it reflects the short-term clinical outcome of a single patient. The pre- and post-treatment color change was not objectively assessed using a standardized shade guide, spectrophotometer, or colorimeter. Esthetic improvement was evaluated only through clinical observation and photographs, which may have been influenced by lighting conditions and camera settings, thereby limiting the objective quantification of color change. Similarly, changes in symptoms were assessed based on the patient’s subjective report and clinical percussion testing, without the use of a standardized pain scale. Long-term clinical follow-up is required to evaluate color stability, restorative success, and possible complications after internal bleaching.

## Conclusions

This case demonstrates that, in symptomatic and discolored endodontically treated anterior teeth, initial control of endodontic infection followed by esthetic rehabilitation with internal bleaching may be applied as a conservative treatment approach. In internal bleaching procedures, proper placement of the cervical barrier, controlled application of the bleaching agent, and appropriate timing of the definitive restoration are important for the biological safety and esthetic success of the treatment. Nevertheless, long-term clinical follow-up is required to evaluate color stability, restorative success, and possible complications.
